# Development of the WHO eye care competency framework

**DOI:** 10.1186/s12960-023-00834-4

**Published:** 2023-06-19

**Authors:** Mitasha Yu, Stuart Keel, Silvio Mariotti, Jody-Anne Mills, Andreas Müller

**Affiliations:** Department of Noncommunicable Diseases, World Health Organization, Geneva, Switzerland

**Keywords:** Eye, Eye care, Competency, Framework, Workforce

## Abstract

**Background:**

The eye care workforce, particularly in lower resource settings, face challenges of limited integration into the health system, limited workforce capacity, mismatch of workforce to population need and poor quality of care. In recognition of these challenges, coupled with a gap in existing tools, provides a strong rationale for the development of the Eye care competency framework (ECCF).

**Methods:**

A mixed methods approach was utilised to develop and validate the ECCF. Content was developed by extracting relevant components of existing frameworks used both within and outside of eye care. A diverse technical working group provided feedback and guidance on the structure, design, and content to create a preliminary draft. Competencies and activities were validated using a modified-Delphi study, and the framework was then piloted at four sites to understand how the tool can be implemented in different settings.

**Results:**

The final version of the ECCF included eight outcomes, nine guiding principles, and content of each of the key elements, including the six domains, 22 competencies, 21 activities, 193 behaviours and 234 tasks, and the knowledge and skills that underpin them. 95/112 participants from the six WHO regions completed the modified-Delphi study, yielding an average of 96% agreement across the competencies and activities in the ECCF. The pilot showcased the versatility and flexibility of the ECCF, where each of the four sites had a different experience in implementing the ECCF. All sites found that the ECCF enabled them to identify gaps within their current workforce documentation.

**Conclusions:**

The ECCF was developed using a collaborative approach, reflecting the opinions of participants and stakeholders from all around the world. The comprehensive competencies and activities developed in the ECCF encompass the diverse roles of eye care workers, and thus encourage multi-disciplinary care and better integration into the health system. It is recommended that eye care workforce planners and developers use the ECCF, and adapt it to their context, to support workforce development and focus on the quality and scope of eye care service provision.

## Background

Globally, at least 2.2 billion people have a vision impairment or blindness, of whom at least 1 billion have a vision impairment that could have been prevented or has yet to be addressed. The eye care workforce, particularly in limited resource settings, face challenges of limited integration into the health system, limited workforce capacity, mismatch of workforce to population need and poor quality of care [1]. A key strategy to address the burden of vision impairment is to improve the eye care workforce’s availability, accessibly and quality, to meet the eye care needs of the population [2].

The tools available for workforce planning and development in eye care globally have been limited [3]. Tools that do exist, commonly support the setting of workforce targets [4–6], rather than supporting workforce development, including the quality and scope of eye care services provision. While popular, the approach has limitations as it does not take into account factors, such as the location and distribution of the current workforce, the population structure, disease epidemiology and the public demand, service regulations and importantly, quality standards [7–9]. In addition, they often isolate the specific occupational groups, further limiting multi-disciplinary care and the integration of eye care into the health system.

A competency framework can be defined as a model that communicates the expected performance and activities of the health workforce across professions, and specializations, to enable quality care and service delivery [10]. They provide a useful tool for workforce planning and development, and serve various roles including outlining characteristics of a competent workforce, facilitating mobility, and analysing or assessing expertise [11]. While competency frameworks have been widely used in many fields, it is important to acknowledge their limitations and consider the applicability of such frameworks in different settings. Competency frameworks do not directly calculate workforce targets; however, they still offer benefits by emphasizing the necessary quality of care and scope of practice to meet the eye care needs of the population. Ultimately, competency frameworks are a tool to strengthen the alignment of the workforce with population needs.

A review of 52 existing competency frameworks and models used in eye care established the lack of a comprehensive global workforce competency framework that encompasses all competencies and activities relevant to eye care [3]. In 2022, Yu et al. proposed the need for a competency framework that encapsulates the roles of the diverse eye care workforce while providing a harmonized language to cut across the occupational groups in eye care and be better integrated into the health system. The value of such a framework would be greatest when designed to be applied to any context, particularly to low–middle income countries, where tools for workforce development are limited [3]. This paper presents the development process of the World Health Organization (WHO) Eye care competency framework (ECCF) [7], including the collaborative content creation, validation and pilot of the ECCF.

## Methods

The study design approach was a mixed methods study conducted in an exploratory sequential design, where the qualitative component was conducted first, followed by the quantitative component. The qualitative component involved collaborative development of the ECCF through a technical working group (TWG) and the creation of the preliminary framework, while the quantitative component involved validation of the competencies and activities through a modified-Delphi study and piloting of the framework at four different sites.

### Collaborative development

To ensure the development of the ECCF was collaborative, international eye care organisations and professional associations were involved and collaborated in a TWG (*n* = 14). The appropriate candidates were selected based on their experience, geographic location, and the occupational group they represented. The TWG comprised of 14 experts (Male: 5, Female: 9) from different countries across the six WHO regions. They represented different occupational groups in eye care: ophthalmology; optometry; and allied ophthalmic personnel (AOP) (Table 1). Their expertise included in-depth knowledge of the eye care sector with experience in workforce planning and development, and integration into health systems. The use of a TWG was necessary to ensure that the perspectives of each occupational group and of different geographic areas and cultures were considered in the development of the ECCF. The TWG was engaged over a 12-month period from April 2021 to April 2022.

**Table 1:** Demographic characteristics represented in the ECCF TWG

Description	Frequency	Percentage (%)
Geographic location by WHO region		
African region	3	21
Eastern–Mediterranean region	2	14
European Region	2	14
Region of the Americas	3	21
South–East Asian Region	1	7
Western Pacific Region	3	21
Location based on economic classification		
Low income countries	1	7
Low–middle income countries	5	36
Upper-middle income countries	2	14
High income countries	6	43
Sex		
Female	9	64
Male	5	36
Occupational group		
Ophthalmologist	5	36
Optometrist	5	36
AOP (e.g., Ophthalmic nurse, Orthoptist)	4	29

### Framework design and structural formation

To create the blueprint and outline of content areas for the new framework, existing frameworks, models and scopes of practice relevant to eye care were collated and analysed, including recognised frameworks used in the health sector. The proposed framework outline was presented to the TWG, who provided feedback that helped shape its design, and the final structure was then collectively approved [3].

### Content formation of the preliminary framework

The preliminary draft of the ECCF was developed through identification of competencies, activities, knowledge and skills drawn from existing competency frameworks, models and scopes of practice relevant to eye care. Content from the existing frameworks was extracted, organised and incorporated into the approved outline. The outcomes and guiding principles were also created to give purpose and guidance (respectively) to the ECCF. The lead author (M.Y.), who was the project leader, reviewed the preliminary draft with TWG through a consultative process, where feedback was received via email communication. After the TWG reviewed the outcomes, the guiding principles, and the key elements of the ECCF, including the competencies, activities, knowledge and skills, feedback was collated, analysed and incorporated in the draft. Any further changes discussed during the subsequent online video meeting were also integrated.

### Validation of the competencies and activities

During a 2-month period from September to October 2021, a modified-Delphi study was carried out to build consensus among a wider group of global eye care workers on the competencies and activities of the preliminary framework. The classic Delphi method was modified to employ binary responses (agree/disagree) to decide whether the statements should be kept or changed, paired with a free-text comment box to note what changes were required to reach the best agreement [10]. The modified-Delphi study was implemented through Survey Monkey and participants were given 4 weeks to complete the survey. The snowball sampling method was used to recruit participants that were identified and referred by the TWG members and were selected based on a similar criterion to the TWG. Participants were asked whether they agreed or disagreed with each competency, activity, the behaviours and tasks expressed across four levels of proficiency, and corresponding knowledge and skills. They were also asked to provide a comment under each competency and activity. Results of the modified-Delphi study was analysed through extracting the data from Survey Monkey and importing it into an Excel spreadsheet. Each comment was inputted line by line against each competency and activity to ensure accurate categorization and tracking of feedback. This systematic approach allowed for a comprehensive and detailed analysis of the data. A review and analysis of the data were conducted to identify common themes and patterns within the feedback. All feedback was considered and integrated into the validated version of the preliminary framework. The results of the modified-Delphi study were shared with the TWG.

### Progress to the final framework

The validated preliminary framework underwent additional development to include the explanatory text. The framework underwent graphical design, particularly to format the complex and extensive tables containing the competencies and activities. To ensure the framework’s comprehensiveness and relevance to the specific context of human resource development in the public health eye care sector, key personnel at WHO and the TWG reviewed it. It should be noted that during this stage, the key components of the ECCF remained unchanged. However, the review process helped refine explanatory text and design of the framework, which led to the penultimate version of the ECCF.

### Piloting the framework

Eye care workforce developers at four sites were consulted over a period of 5 months, to conduct a pilot on the implementation of the ECCF. The sites were identified through members of the TWG with the criteria being that each provided a different setting, i.e., tertiary eye hospital setting; professional body representing a specific occupational group locally and globally, and teaching institutes. Each site also varied from a low–middle income to a high-income setting. The aim of the pilot was to have a clearer understanding of how the ECCF would be utilised in each setting, and to identify any additional resources required to assist in the application of the ECCF. Following the completion of the pilot, each site was asked to complete a brief questionnaire to detail the process of how they applied the framework, and to identify any lessons learnt.

## Results

### Construction of preliminary framework

**Fig. 1:**
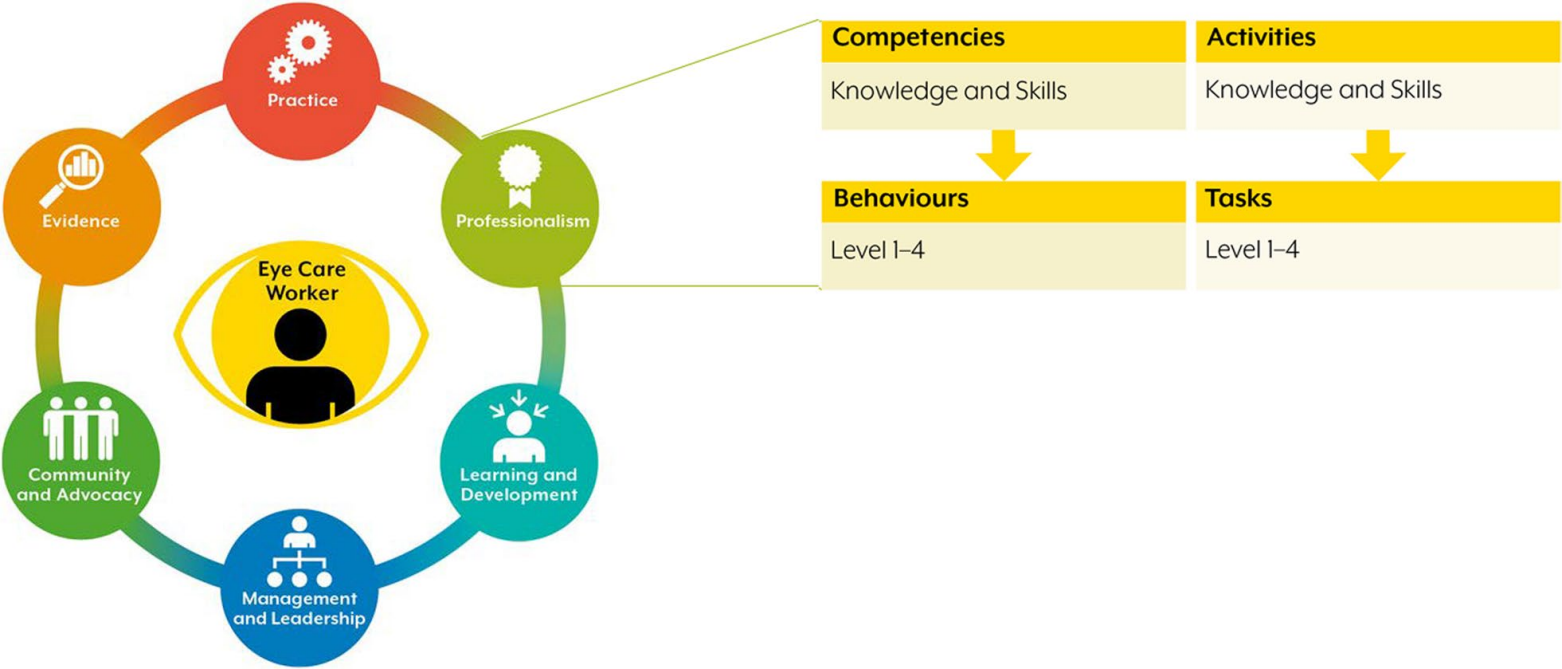
The structural arrangement of the ECCF comprised of six domains, each accompanied by competencies and activities

The preliminary framework included the construction of the outcomes, the guiding principles, and the key elements of the ECCF, including the domains, competencies, activities, knowledge and skills. The ECCF presents the competencies and activities that encompass the eye care worker through six domains: Practice, Professionalism, Learning and Development, Management and Leadership, Community and Advocacy, and Evidence [3]. Within these domains, knowledge and skills underpin each competency and activity. Competencies are described through behaviours and applicable to all eye care workers, regardless of their scope of practice. On the other hand, activities are described through tasks, and are selectively applicable to eye care workers, depending on their scope of practice within their specific context. Behaviours and tasks for each competency and activity (respectively) are expressed at four different levels of proficiency (Level 1: Introductory; Level 2: Intermediate; Level 3: Advanced; and Level 4: Expert) (Fig. 1). The levels represent an increase in autonomy and decision making and enable the ECCF to be applied across a diverse eye care workforce. In total there were 22 competencies, 21 activities, 193 behaviours and 234 tasks that were developed into the ECCF.

The eight outcomes for the ECCF that were developed clearly highlight the aims of the framework (Table 2). These outcomes will illustrate to workforce planners how the ECCF can assist in planning, and potentially be used to advocate for the implementation of the ECCF within a setting. Nine guiding principles (Table 3) of the ECCF were adopted and adapted from the WHO Global strategy on human resources for health 2030 [2].

**Table 2:** Eight outcomes of the ECCF

1. Establishing a foundation for workforce planning and development to inform education institutions in preparing workers for practice
2. Guiding overarching practice standards for practitioners, employers, policy makers and regulatory bodies to assist in recruitment, employment, appraisals and regulation
3. Defining a shared set of expectations with common language to support alignment between education and employment
4. Focusing on what competencies and activities are required to provide eye care services to meet the need, rather than a focus on who should provide them
5. Bringing attention to the continuum of eye care across all levels of the health system, particularly primary eye care, to support Universal Health Coverage
6. Giving flexibility and enabling the ECCF to be tailored and adapted to suit different contexts and allowing room for individuals to participate in lifelong learning
7. Promoting collaboration among eye care disciplines and specializations through harmonized language and structure
8. Providing a strong public statement about the importance of integrated and people-centred eye care workforce

**Table 3:** Nine guiding principles of the ECCF

1. Promote the right to the enjoyment of the highest attainable standard of health
2. Provide integrated, people-centred health services devoid of stigma and discrimination
3. Foster empowered and engaged communities
4. Uphold the personal, employment and professional rights of all health workers including safe and decent working environments and freedom from all kinds of discrimination, coercion, and violence
5. Eliminate gender-based violence, discrimination, and harassment
6. Promote disability inclusiveness in all their diversity
7. Promote collaboration and solidarity in alignment with national priorities, and integration with health systems
8. Ensure ethical recruitment practices in conformity with the provisions of the WHO Global Code of Practice on the International Recruitment of Health Personnel [12]
9. Promote innovation and the use of evidence

### Validation process: a modified-Delphi study

**Fig. 2:**
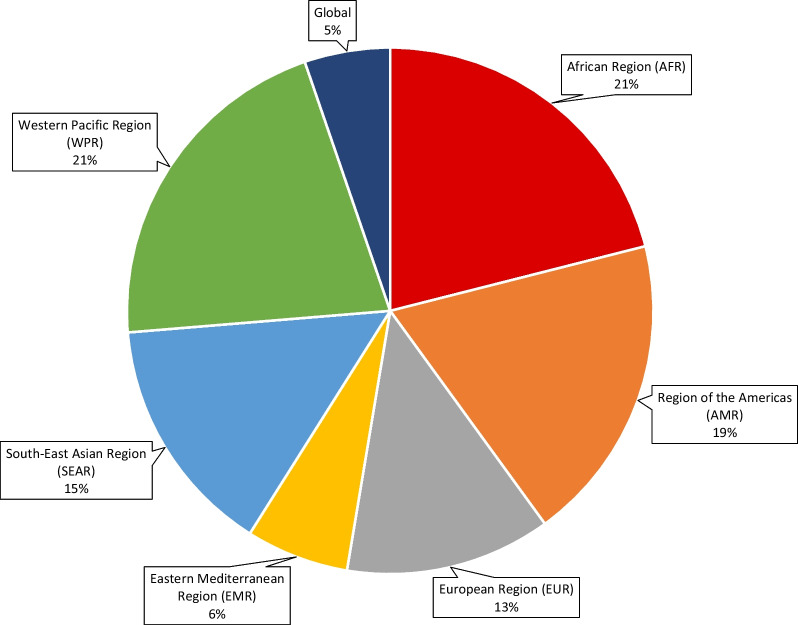
Geographic location of participants of the modified-Delphi study, by WHO region

Of the 112 participants invited to participate in the modified-Delphi study, 95 (85%) completed the study. The gender distribution was female 51.6%. The participants comprised of eye care practitioners, educators, managers, researchers, policy makers and programme implementers. There was representation of participants from all six of the WHO geographic locations, with the highest from African region and Region of Americas, each at 20.7%, and the lowest representation from Eastern Mediterranean Region at 7.3% (Fig. 2).

The participants also represented several occupational groups within eye care, ophthalmology 40.2%, optometry 46.3%, AOP/mid-level eye care personnel 15.5%, vision technician/refractionist 3.7%, and community eye care worker/community health worker 1.2%.

Of the participants, 59.8% stated that they were highly involved in eye care workforce planning and development, and 62.2% of participants stated they had a strong ability to implement and/or influenced competencies for eye care workers within their respective settings.

**Fig. 3:**
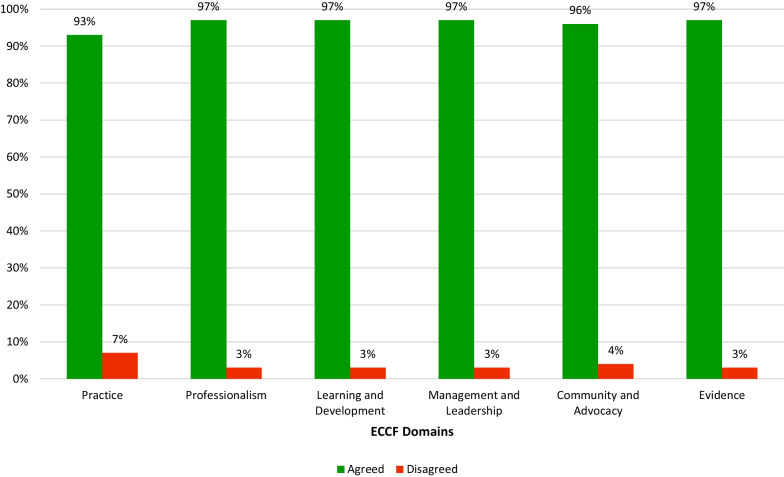
Percentages of agreement and disagreement across the competencies and activities of six domains

The study participants reviewed and provided their feedback on the key components of the ECCF, eliciting approximately 3000 comments. Most of the feedback received from the participants focused on the complexity of the language used in the framework, with many suggesting that it could be simplified further to enhance its accessibility and usefulness. The Practice domain was a particular area of contention, with some participants expressing disagreement with the categorization of eye care interventions across the different levels of proficiency. There was an average of 96% agreement across the competencies and activities in the ECCF (Fig. 3). The study was conducted in one round, given the high agreement percentage which was well above the target of 80% agreement [13–15]. All results, including comments, were reviewed with the TWG, and minor amendments were made.

### Formulation of the final framework

The final framework included the development of the explanatory text, such as the executive summary; background and rationale; overview of the ECCF and its components; definitions; and description of the eye care workforce. The outcomes, the guiding principles, and the key elements of the ECCF, including the domains, competencies, activities (Table 4), knowledge and skills, were also finalised. These elements combined to establish the ECCF. The graphical design process presented the ECCF in format that made it easier for users to read.

**Table 4:** List of the competencies and activities across the six domains

Practice domain (P)	
	Competency (C)
PC1	Maintains people-centred practice
PC2	Performs within scope of practice and abilities
PC3	Applies current evidence-based best practice appropriate to context
PC4	Applies a rational approach to problem-solving and decision-making
PC5	Communicates effectively with a person, their family and carers
	Activity (A)
PA1	Obtaining informed consent
PA2	Maintaining documentation
PA3	Conducting vision assessment and eye examination
PA4	Establishing a diagnosis
PA5	Providing information and advice to a person, their family and carers
PA6	Managing referrals
PA7	Establishing collaborative eye care management plans
PA8	Conducting eye care interventions
PA9	Ensuring continuity of care
Professionalism domain (PM)	
	Competency (C)
PMC1	Practices professional and ethical conduct
PMC2	Practices within the legal and/or regulatory framework
PMC3	Manages professional responsibilities
PMC4	Demonstrates awareness and responsiveness to intersectionality, socioeconomic and environmental factors
PMC5	Appropriately represents the role of eye care workers
	Activity (A)
PMA1	Managing risks
PMA2	Improving quality
PMA3	Implementing inclusive practice
Learning and Development domain (LD)	
	Competency (C)
LDC1	Maintains learning and development
LDC2	Supports others to learn and develop
LDC3	Strengthens educational training capacity in eye care
	Activity (A)
LDA1	Continuing education
LDA2	Developing and teaching others
Management and Leadership domain (ML)	
	Competency (C)
MLC1	Enhances the eye care team
MLC2	Enhances eye care service development
MLC3	Integrates eye care services as part of universal health coverage
	Activity (A)
MLA1	Managing an eye care team
MLA2	Managing eye care service delivery
Community and Advocacy domain (CA)	
	Competency (C)
CAC1	Supports integrated people-centred eye care (IPEC) in health systems
CAC2	Empowers the community
CAC3	Enhances community awareness and health promotion
CAC4	Acts as an advocate for eye care
	Activity (A)
CAA1	Developing integrated people-centred eye care (IPEC) plans and programmes
CAA2	Disseminating relevant health promotion messages
Evidence Domain	
	Competency (C)
EC1	Integrates evidence-based practice
EC2	Strengthens research capacity in eye care
	Activity (A)
EA1	Planning and implementing research
EA2	Disseminating evidence
EA3	Monitoring and evaluation

### Implementation of the ECCF: a pilot study

The pilot was conducted at four sites, each showcasing a unique context illustrating the versatility and flexibility of the ECCF. At all the pilot sites, the ECCF enabled the identification of gaps within the existing workforce documentation, such as the topics in the curriculum, or the competencies expected from the current workforce. The pilot highlighted the need to adapt the ECCF to make it suitable for the context and identified the importance for an accompanying guidance document to assist users of the ECCF. The questionnaire following the completion of the pilot, yielded lessons learnt from each site (Table 5).

**Table 5:** Lessons learnt from each of the pilot sites [16]

Application description	Lessons learnt
Pilot site 1: Aravind Eye Care, India (Aravind)	
Workforce review in a hospital setting: Aravind used the ECCF to evaluate the competencies of the current workforce it employs	• The ECCF displays competencies at a high level, but it also enables a careful assessment of the skills and tasks expected of the eye care personnel • The ECCF enabled Aravind to reflect on developing the aspirational leadership qualities for each “leader” staff member. It highlighted the need to have a structured training to holistically develop an ophthalmologist or AOP into a leader, particularly in the non-clinical domains, such as Evidence, Community and advocacy and Professionalism. Thus, key performance indicators and other methods for staff management should take into consideration for leadership development • The ECCF can be used more effectively if it is simplified and contextualised
Pilot site 2: L’Ordre des Optométristes du Québec, Canada (OOQ)	
Development of a professional body’s competency framework: the OOQ re-evaluated their existing competency framework using the ECCF to analyse if it reflected both current practices and best practices	• The existing competency framework had limited integration of the target workforce in the health care system. By identifying these gaps and adopting relevant competencies and activities from the ECCF that addressed this, the amended competency framework can now work towards a target workforce that has better integration within the health care system • Competency frameworks are often complex and challenging for people unfamiliar with them to navigate. For people wanting to develop their own competency framework, it is helpful for them to have a solid understanding of the concept of a competency framework, before embarking on developing one • During the adaptation process, the approach of researching best practices was used to resolve opposing feedback
Pilot site 3: International Joint Commission on Allied Health Professionals in Ophthalmology (IJCAHPO) in partnership with L V Prasad Eye Institute, India (LVPEI)	
Development of professional body’s competency-based framework and curriculum: the ECCF was adapted and aligned to the IJCAHPO’s standards for education and training, certification and training programme accreditation of AOP; further application of the of the ECCF was made to LVPEI’s local ophthalmic practices in their training and education	• The ECCF enabled the need for focus on several non-clinical aspects such as professionalism and learning at the study site which were not emphasized in our previous curriculum. In addition, the pilot made IJCAHPO recognize they could assist other organizations working with AOPs to apply the ECCF to their training programmes • Evidence-based practice is more emphasized in advanced programmes than in the IJCAHPO’s syllabus. This pilot study identified areas to focus on to bring evidence-based practice to the AOP/vision technician curriculum • Although there was an existing programme, the alignment of the programme to the ECCF allowed for a structured and comprehensive competency approach to curriculum development • Several gap areas were identified in the existing curriculum, such as quality assurance, integration of a patient-centred approach, application of a rational approach to problem-solving and decision making, and establishment of a collaborative eye care management plan • The IJCAHPO curriculum had the opportunity now to include more components of research through the integration of the evidence domain from the ECCF
Pilot site 4: Singapore National Eye Centre	
Development of a competency-based curriculum: to apply the ECCF to an existing training programme, enabling the benchmarking of the selected curriculum against the ECCF	• It is important to consider the learning goals/objectives of the programme when implementing the ECCF, as not every competency and activity may be relevant, for example, leadership competencies for a basic upskill programme • An iterative process should be maintained when designing the programme/curriculum using the ECCF, including review, monitoring and evaluation of outcomes to garner feedback from all stakeholders, and then return to improve the curriculum • With existing programmes, it may be easier first to adapt the ECCF and then apply it rather than trying to apply an existing training programme to the full ECCF • The ECCF serves as a reminder that non-clinical aspects of the programme are equally important to ensure the holistic development of an eye care professional • There is potential for the adapted ECCF to be used for advocacy at the ministry/policy level, and other workforce areas such a service delivery facility at local and regional hospitals • It is possible to determine whether the eye care system can produce personnel with the necessary expertise to deliver comprehensive eye care services within the health system, by outlining the competencies and activities of each position and a corresponding education/development plan in conjunction with human resources

## Discussion

A mixed methods approach was used to produce the ECCF, combining both qualitative and quantitative data. Maximum efforts were made to ensure that viewpoints and perspectives from a variety of frameworks, occupational groups, and people from around the world were included in the development process of the ECCF, because it is intended to be a tool that is universally applicable for the eye care workforce globally. The diverse viewpoints gained from the collaborative approach, further directed the development of the guiding principles, which serve to define the values and tenets of the framework. It is envisaged that these principles will be extended into the workforce, where the ECCF is being implemented.

Despite the fact the eye care sector is relatively mature in many countries, the adoption of competency frameworks is still novel. The use of the ECCF can be particularly beneficial in settings where the eye care workforce is still emerging or in instances where there is a labour supply shortage, and tasks need to be distributed efficiently and effectively to optimise access to care [10, 15]. This aspect was taken into account when developing the ECCF, whose key components encompassed the behaviours and tasks expressed over four levels of proficiencies. The TWG was instrumental in guiding and determining how the behaviours and tasks were described across the four levels of proficiency, to capture the scope of performance represented in the eye care workforce. The knowledge and skills included were not exhaustive, but rather a sample suggestive list for users of the ECCF to consider and further expand in their own contexts.

The Delphi technique was chosen for this study, because it is widely used to develop consensus on group opinion and is frequently used in the development of competency frameworks for personnel in a particular field [8, 18]. While the modified-Delphi technique used in this study deviated from traditional approaches in some ways, author’s acknowledged the importance of its foundational principles, including anonymity, iteration, regulated feedback, statistical group response, and structure [19, 20]. In particular, the study aimed to address issues such as groupthink and dominance bias that can arise in face-to-face group meetings, by conducting the survey online. It should be noted that the study only utilized one consensus approach.

In addition, the modified-Delphi survey consisted of participants entering in the responses to questions asked in a binary format. Binary responses were preferred than other formats, such as a Likert scale, as they captured the opinions of the participants in a straightforward and concise manner, and this approach has been shown to be effective in previous research [10, 21–23]. Furthermore, as the survey was based on the comprehensive competency framework and consisted of 265 questions, the use of binary responses was advantageous in that it allowed participants to complete the lengthy survey with greater ease and efficiency.

The snowball sampling method was used to recruit participants for the modified-Delphi survey, relying on referrals from existing TWG members. This approach was found to be effective, as many of the TWGs were part of professional associations and networks with extensive reach and connections within the public health eye care sector, facilitating the identification of a diverse group of individuals with the necessary expertise for the survey [24]. To reach these individuals, other recruitment methods such as open call outs through social media or other platforms would have been less effective due to the limited pool of qualified individuals and the potential challenges in reaching them. Therefore, targeting these individuals through a snowball sampling method was a decision that produced favourable results within the project timeline.

The high agreement percentage (96% agreement) of the modified-Delphi study may be attributed to several factors. First, the study focused on a narrow and specific topic, which made it easier for the participants to reach a consensus. Second, the participants were selected based on their expertise and experience in the field, which ensured that they had a good understanding of the issues and were able to provide informed responses. Third, the binary response format simplified the decision-making process and eliminated the ambiguity that often arises in rating scales. The reasons for disagreements that arose was shown in the comments section. The few points of disagreement that surfaced in the comments section were analysed and revealed that the Practice domain was an area, where participants had slightly diverging opinions. Specifically, some participants expressed dissatisfaction with the categorization of eye care interventions across different proficiency levels, citing inadequate definition and differentiation. Others argued that certain interventions could be classified into multiple categories. Similar concerns were raised during the TWG discussions in the development of the draft, and such comments were expected in the modified-Delphi survey, given the intentional broad scope of the competency framework to make it relevant and usable globally. Despite these disagreements, the feedback overall was constructive and valuable in assisting to refine and improve the framework.

The pilot process was beneficial for testing the ECCF in a practical environment. The common result of each site identifying gaps within the current workforce documentation can be attributed to the comprehensive nature of the ECCF, which has been limited in previous workforce planning tools within eye care. Each site’s pilot experience was different, and each site applied the ECCF differently. It is crucial to acknowledge that the ECCF, like all competency frameworks, cannot be applied universally and must be tailored to meet the specific needs and context of each setting. Moreover, it is important to acknowledge that competency frameworks are not a one-size-fits-all solution, and further discussion and critique on their validity and limitations in different contexts, including in eye care, would be beneficial for advancing their development and use.

The pilot highlighted the need for a guidance paper to make it easier for users to adapt and apply the ECCF in their settings. It was recommended that the learnings from the pilot process be converted to case studies, as it would be beneficial to show potential users on how they could apply the ECCF in their setting. A further recommendation would be to include these case studies within the guidance document that also shows users, a step-by-step process on how to adopt and adapt the ECCF for their individual settings. In addition, the pilot process gave valuable insights on how the ECCF can be used in conjunction with other existing approaches in workforce planning and development. By emphasizing the necessary competencies and activities required for high-quality comprehensive eye care services, the ECCF can serve as a valuable reference tool for improving workforce quality and integrating eye care services into the broader health system.

The development of the ECCF aims to facilitate eye care workforce planning and development by providing a comprehensive set of competencies and activities that encompass the diverse roles represented by eye care workers. This paper complements already published ECCF materials [7, 16] by presenting the thoroughness of the development process, and rationale for an accompanying guidance document. Given the lack of eye care workforce tools particularly for low–middle income resource settings and multi-disciplinary services [3], the ECCF can be a useful reference tool to assist in identifying gaps in current education programmes, workforce planning documentation, and frameworks used to describe the competencies for occupational groups within eye care. For the ECCF to be implemented successfully, a variety of stakeholders, including governments, the WHO, non-governmental organisations, and the private sector, will need to work together to invest in eye care workforce development while also establishing stronger links between eye care education programmes, service providers, and regulatory bodies.

### Limitations

Users of the ECCF should consider four key limitations when applying the framework. First, an extensive range of eye care professionals from a variety of nations, including both high- and low-income settings, were represented among the study's stakeholders and participants. Despite this, there were still only small number of participants representing the East Mediterranean region in the modified-Delphi study which may result in some components of the framework not applicable to the workforce in this region. Second, although the ECCF is targeted at both government and non-government institutions, a government setting was not selected for the pilot, as it would have been challenging to implement within the project timeline. Given this limitation, it might be more difficult to understand how the framework can be applied in a government setting. Third, the recruitment of participants for the modified-Delphi study was carried out using the snowball sampling method, where referrals were made from the TWG. Although this assisted in reaching the target audience within the study’s time frame, this may have introduced selection bias. Fourth, the use of binary responses and the increased length of the modified-Delphi survey may have introduced limitations to the study. The binary response options may have led to oversimplification of responses, potentially resulting in less nuanced feedback. In addition, the length of the survey may have led to participant fatigue and rushed responses, potentially affecting the quality of the data collected. These limitations should be considered when interpreting the study findings.

## Conclusions

A mixed methods study was used in the development of the ECCF. This approach was chosen as it aligned to views of stakeholders and participants from across the world, including workforce experts from the diverse occupational groups found within eye care, which further enabled the ECCF to have a harmonized language to cut across these groups. The pilot provided an opportunity to learn from applying the ECCF in a practical situation and determine how to use it most effectively. An accompanying guidance document to the ECCF would be beneficial in its implementation as it will provide a step-by-step guidance on how to adapt and adopt the ECCF to make it suitable to each setting [16]. Planners and educators of the eye care workforce are encouraged to use the ECCF to ensure they include the diverse competencies and activities of eye care workers within their programmes, so that the workforce in the eye care sector is in line with population need.

## Data Availability

Raw data from the findings are available on request. Contact: miyu@who.int.

## Abbreviations

ECCF: Eye care competency framework
TWG: Technical working group
WHO: World Health Organization
AOP: Allied ophthalmic personnel

## References

[1] World Health Organization. World report on vision [Internet]. Vol. 214, World health Organization. 2019. 1–160 p. https://www.who.int/publications/i/item/9789241516570. Accessed 23 Jan 2023.

[2] World Health Organization. Global strategy on human resources for health: Workforce 2030 [Internet]. World Health Organization. 2016. https://apps.who.int/iris/bitstream/handle/10665/250368/9789241511131-eng.pdf. Accessed 23 Jan 2023.

[3] Yu M, Keel S, Mills A, Müller A (2022). Investigating the need and structure for a comprehensive eye care competency framework. BMJ Open Ophthalmol..

[4] Ali AA, Hallingham S, Buys YM (2015). Workforce supply of eye care providers in Canada: optometrists, ophthalmologists, and subspecialty ophthalmologists. Can J Ophthalmol.

[5] Feng PW, Ahluwalia A, Feng H, Adelman RA (2020). National trends in the United States eye care workforce from 1995 to 2017. Am J Ophthalmol.

[6] Resnikoff S, Lansingh VC, Washburn L, Felch W, Gauthier TM, Taylor HR (2020). Estimated number of ophthalmologists worldwide (International Council of Ophthalmology update): will we meet the needs?. Br J Ophthalmol [Internet].

[7] World Health Organization. Eye care competency framework [Internet]. 2022. https://www.who.int/publications/i/item/9789240048416. Accessed 18 Jan 2023.

[8] Shah K, Naidoo K, Loughman J (2016). Development of socially responsive competency frameworks for ophthalmic technicians and optometrists in Mozambique. Clin Exp Optom.

[9] Shah T, Milosavljevic S, Bath B (2020). Geographic availability to optometry services across Canada: mapping distribution, need and self-reported use. BMC Health Serv Res.

[10] Mills J, Cieza A, Short SD, Middleton JW (2021). Development and validation of the WHO rehabilitation competency framework: a mixed methods study. Arch Phys Med Rehabil [Internet].

[11] Batt AM, Tavares W, Williams B. The development of competency frameworks in healthcare professions: a scoping review, Vol. 25, Advances in Health Sciences Education. Springer; 2020. p. 913–87. 10.1007/s10459-019-09946-w 31797195

[12] Dayrit M, Taylor A, Yan J, Braichet JM, Zurn P, Shainblum E (2008). WHO code of practice on the international recruitment of health personnel. Bull World Health Organ.

[13] Eubank B, Mohtadi N, Lafave M, Wiley J, Bois A, Boorman R (2016). Using the modified Delphi method to establish clinical consensus for the diagnosis and treatment of patients with rotator cuff pathology. BMC Med Res Methodol.

[14] Stewart D, Gibson-Smith K, MacLure K, Mair A, Alonso A, Codina C (2017). A modified Delphi study to determine the level of consensus across the European Union on the structures, processes and desired outcomes of the management of polypharmacy in older people. PLoS ONE.

[15] Snape D, Kirkham J, Preston J, Popay J, Britten N, Collins M (2014). Exploring areas of consensus and conflict around values underpinning public involvement in health and social care research: a modified Delphi study. BMJ Open.

[16] World Health Organization. Guide to applying the WHO Eye Care Competency Framework [Internet]. World Health Organization; 2022. https://www.who.int/publications/i/item/9789240061422. Accessed 2 Feb 2023.

[17] Liu JX, Goryakin Y, Maeda A, Bruckner T, Scheffler R (2017). Global health workforce labor market projections for 2030. Hum Resour Health.

[18] Trevelyan EG, Robinson N (2015). Delphi methodology in health research: how to do it?. Eur J Integr Med.

[19] Nasa P, Jain R, Juneja D (2021). Delphi methodology in healthcare research: How to decide its appropriateness. World J Methodol.

[20] Humphrey-Murto S, Varpio L, Wood TJ, Gonsalves C, Ufholz LA, Mascioli K (2017). The use of the delphi and other consensus group methods in medical education research: a review. Acad Med.

[21] Galesic M, Bosnjak M (2009). Effects of questionnaire length on participation and indicators of response quality in a web survey. Public Opin Q.

[22] Dolnicar S, Grün B, Leisch F (2011). Quick, simple and reliable: forced binary survey questions. Int J Mark Res.

[23] Grassi M, Nucera A, Zanolin E, Omenaas E, Anto JM, Leynaert B (2007). Performance comparison of likert and binary formats of SF-36 version 1.6 across ECRHS II adults populations. Value Heal..

[24] Valerio MA, Rodriguez N, Winkler P, Lopez J, Dennison M, Liang Y (2016). Comparing two sampling methods to engage hard-to-reach communities in research priority setting. BMC Med Res Methodol.

